# Non-Celiac Gluten Sensitivity in the Context of Functional Gastrointestinal Disorders

**DOI:** 10.3390/nu12123735

**Published:** 2020-12-04

**Authors:** Maria Raffaella Barbaro, Cesare Cremon, Diana Wrona, Daniele Fuschi, Giovanni Marasco, Vincenzo Stanghellini, Giovanni Barbara

**Affiliations:** 1 IRCCS S. Orsola, 40138 Bologna, Italy; maria.barbaro2@unibo.it (M.R.B.); cesare.cremon@aosp.bo.it (C.C.); dianawrona@hotmail.it (D.W.); daniele.fuschi2@unibo.it (D.F.); giovannimarasco89@gmail.com (G.M.); v.stanghellini@unibo.it (V.S.); 2 Department of Medical and Surgical Sciences, University of Bologna, 40138 Bologna, Italy

**Keywords:** functional gastrointestinal disorders, disorders of gut-brain interaction, diet, gluten, IBS, DGBI, NCGS

## Abstract

Gluten-free diets are increasingly chosen in the Western world, even in the absence of a diagnosis of celiac disease. Around 10% of people worldwide self-report gluten-related complaints, including intestinal and extra-intestinal symptoms. In most cases, these subjects would be labeled as patients suffering from irritable bowel syndrome (IBS) who place themselves on a gluten-free diet even in the absence of celiac disease. In some instances, patients report a clear benefit by avoiding gluten from their diet and/or symptom worsening upon gluten reintroduction. This clinical entity has been termed non-celiac gluten sensitivity (NCGS). The symptoms referred by these patients are both intestinal and extra-intestinal, suggesting that similarly to functional gastrointestinal disorders, NCGS is a disorder of gut–brain interaction. It remains unclear if gluten is the only wheat component involved in NCGS. The mechanisms underlying symptom generation in NCGS remain to be fully clarified, although in the past few years, the research has significantly moved forward with new data linking NCGS to changes in gut motility, permeability and innate immunity. The diagnosis is largely based on the self-reported reaction to gluten by the patient, as there are no available biomarkers, and confirmatory double-blind challenge protocols are unfeasible in daily clinical practice. Some studies suggest that a small proportion of patients with IBS have an intolerance to gluten. However, the benefits of gluten-free or low-gluten diets in non-celiac disease-related conditions are limited, and the long-term consequences of this practice may include nutritional and gut microbiota unbalance. Here, we summarize the role of gluten in the clinical features, pathophysiology, and management of NCGS and disorders of gut–brain interaction.

## 1. Introduction

Patients suffering from gastrointestinal complaints often report that symptom onset or worsening occurs with food ingestion and ask for dietary advice from their physicians. Accordingly, up to 84% of patients suffering the irritable bowel syndrome (IBS), which is one of the most common functional gastrointestinal disorders, now called disorders of gut–brain interaction (DGBI), report that symptoms were related to at least one food item [1]. Recently, there has been increasing attention to the role of diets in gastrointestinal disorders. New trends include the adoption of gluten-free, low-carbohydrate, low-fermentable oligosaccharide, disaccharide, monosaccharide, and polyols (FODMAPs), and anti-allergy diets. In this review, we will summarize current concepts related to the increasing attention to gluten-free diets in conditions such as gluten sensitivity and DGBI.

## 2. The Boom of Gluten-Free Diets

While a gluten-free diet is the cornerstone of celiac disease (CD), its use has been extended to numerous other gastrointestinal and extra-intestinal conditions. This new trend is not surprising, considering that the reported global prevalence of health complaints related to gluten consumption ranges between 4% and 15% [2,3,4,5,6,7,8,9,10,11], with a pooled prevalence of approximately 10% [12]. In 2015, the gluten-free food market was worth $11 billion [13], while in 2019 it was estimated around $21.61 billion, and it is expected to further increase between 2020 and 2027 [14].

The vast majority of consumers of gluten-free products buy them for reasons other than CD [15], even if gluten-free diet (GFD) products are considerably more expensive than their gluten-containing foodstuffs and in most countries are not reimbursed for non-CD patients.

According to a market research survey, healthy people declared to purchase gluten-free products for the following reasons: to try something new (35%), believing these products are healthier (30%), for weight control (23%), for a special eating plan or diet (19%), and believing these products taste better (14%); only 21% declare to buy these products for someone else avoiding gluten [16,17].

In addition, GFD has been particularly promoted by celebrities and athletes [16]. In a global survey of 910 athletes, 41% of them declare to adopt a GFD, considering this choice improves their performance [18]. Therefore, being sponsored as a healthy diet, people tend to consume more gluten-free foods [19].

Nonetheless, scientific data report that long-term gluten restrictions can be harmful to health. In fact, GFD, at least in patients with CD, has been associated with changes in cardiovascular risk factors [20], nutritional deficiency of vitamins [21], accumulation of heavy metals such as lead, mercury, and cadmium, as well as elevated urinary concentrations of total arsenic, compared to subjects eating a standard diet [22,23], which has been attributed to increased consumption of fish and rice, both rich in these heavy metals. In addition, GFD is rich in other high-energy compounds compared to a normal diet, such as lipids, sugars, and salt, which can contribute to obesity, dyslipoproteinemia, insulin resistance, or metabolic syndrome [22]. Interestingly, the consumption of GFD has been linked to mood modifications and the development of anxiety [24]. Finally, a double-blind randomized controlled trial in healthy controls demonstrated that gluten consumption did not induce symptoms in healthy subjects [25].

Based on current evidence, a gluten-free diet is mandatory for CD patients, while it remains to be clarified which patients with gluten-related disorders really benefit from adopting it.

## 3. Ancient and Modern Wheats

Recently, there has been a renewed interest in ancient cereals promoted by the search for grains with greater nutritional benefits [26]. A recent review indicates that the nutrient composition is not considerably different between ancient and modern grains, except for more advantageous mineral content in ancient grains [26]. However, the different processes of handling and transforming modern grains compared to that used for ancient grains affect the immunogenic capacity. In addition, ancient diploid grains, in particular *Triticum monococcum*, showed a higher digestibility of proteins, especially gluten and α-amylase/trypsin inhibitors (ATIs), which are known for their pro-inflammatory activity [27,28]. While ancient grains seem to contain lower immunogenic peptides, the heritage grains, i.e., grains introduced during last centuries before the second World War [26], contain equal or even higher toxic peptides compared to modern grains [27]. A study by Ficco et al. evaluated in vitro digestion of nine heritage and three modern grains. The results demonstrated that five immunogenic γ-gliadin peptides were more abundant in heritage compared to modern grains [29].

## 4. Wheat Components and their Impact on Gut Physiology

**Figure 1:**
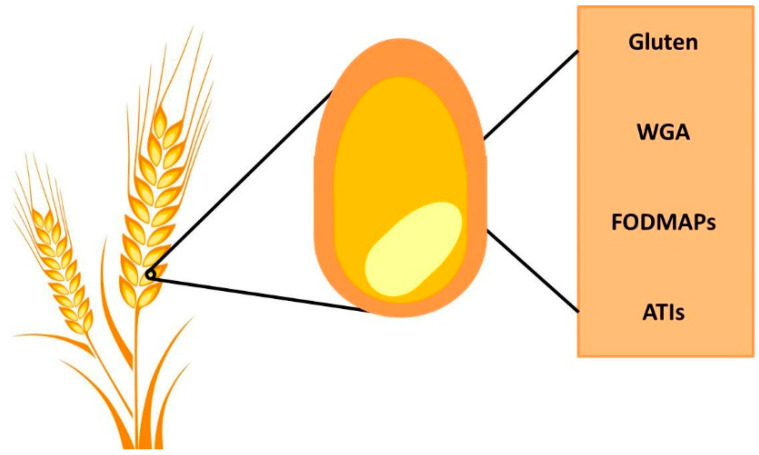
Wheat components. Gluten is the most abundant protein in wheat, but there are also other important components: wheat germ agglutinin (WGA), fermentable oligosaccharides, disaccharides, monosaccharides and polyols (FODMAPs) and α-amylase/trypsin inhibitors (ATIs).

In addition to gluten, wheat contains other components that can have a strong impact on gut physiology, including wheat germ agglutinin (WGA), FODMAPs, and α-amylase/trypsin inhibitors (ATIs) (Figure 1).

### 4.1. Gluten

Wheat is one of the major food crops cultivated and consumed worldwide. The average gluten intake in Western diets is estimated in the range of 5–20 g/day. Gluten proteins are the most abundant proteins in the wheat kernel and are essential for the germination and the development of the seed [30]. Gluten proteins form a network in dough essential to ensure the cohesiveness of wheat required to make products such as bread and pasta. Gluten proteins are prolamins, rich in glutamine and proline, and they have different names, including (a) gliadins (monomers) and glutenins (polymers) in wheat, (b) hordeins in barley, and (c) secalins in rye [31]. The abundance of glutamine and proline in gliadin reduces the breakdown of gluten by proteases in the gastrointestinal tract, hence reducing digestibility. Gliadin has been shown to induce a transient increase in intestinal permeability in healthy subjects, which is directly related to the amount of ingested peptide [32,33]. Although the mechanism underlying this increase in epithelial permeability remains unclear, it has been suggested that a key event is the binding of gliadin to the C-X-C Motif Chemokine Receptor 3 (CXCR3) on epithelial cells. This binding induces an increase in intestinal permeability through a MyD88-dependent release of zonulin. Zonulin-dependent disengaging of tight junctions enables the passage of gliadin through the epithelial barrier into the mucosa [34], ultimately enhancing the passage of indigested peptides into the *lamina propria* followed by overstimulation of the immune system. Glutamine and proline are the substrates of mucosal tissue transglutaminases (tTGs). The peptides produced by tTGs have a great affinity for major histocompatibility complex II (MHC II), which strongly stimulates the immune system in human leukocyte antigen (HLA)-DQ2/8-positive subjects [35]. The prototype of gluten-related disorders is CD, in which the antigen presentation to T cells induces innate and adaptive responses that culminate in villus atrophy, crypt hyperplasia, and enhanced infiltration of intraepithelial lymphocytes [36,37]. A recent study confirmed the correlation between gluten consumption and the risk to develop CD in children [38]. Gut microbiota could play an important role in the pathophysiology of gluten-related disorders [39,40], principally because microbes can digest gluten, forming immunogenic peptides, which activate the immune system. The analysis of urine metabolites by nuclear magnetic resonance showed a characteristic profile in CD patients different from healthy subjects. Interestingly, the metabolites differentiating CD from controls were linked to the gut microbiota, and these differences were abolished after a GFD [41]. Initial evidence suggests that gluten can have an impact on the central nervous system, as well as mood and psychological aspects by changing gut microbiota. Meanwhile, neuritis, ataxia, and partial degeneration of the spinal cord in patients with CD have been known for decades and have been attributed to the central role of tissue transglutaminase-2 (tTG2), which would function as an autoantigen, thereby inducing the formation of tTG2 autoantibodies. The interaction between gluten and microbiota has been recently reported in a randomized double-blind placebo-controlled (DBPC) study which showed that the combination of a GFD and probiotic improved psychiatric symptoms, preventing inflammation and improving the gut barrier [42].

### 4.2. α-Amylase/Trypsin Inhibitors (ATIs)

Four percent of total wheat proteins is represented by ATIs, which activate immune cells, including monocytes, macrophages, and dendritic cells, and induce the release of pro-inflammatory cytokines both in cell lines, human duodenal biopsies, and in mice [43]. Zevallos et al. demonstrated that the modern wheat staple contains a higher amount of ATIs compared to ancient grains. These proteins are highly resistant to proteases and heat. ATIs are potent activators of the Toll-like receptor (TLR)-4-MD2-CD14 pathway resulting in the activation of gut and mesenteric lymph node myeloid cells, finally culminating in intestinal inflammation in mice [44]. Based on this background, ATIs have been suggested to participate in the pathophysiology of non-celiac gluten sensitivity (NCGS) through the activation of innate immunity leading to gut inflammation, although corresponding data in humans are still lacking [44].

### 4.3. FODMAPs

Wheat contains a large amount of carbohydrates, including fructans, which are poorly absorbed by the small bowel, as well as other fermentable oligosaccharides, disaccharides, monosaccharides and polyols, which are collectively grouped under the name of FODMAPs.

FODMAPs are characterized to be fermentable and poorly absorbed, and the shorter molecules are osmotically active [45]. This last characteristic of FODMAPs is responsible for the increase of water in the small bowel and the accelerated transit through it, while the distention of the colon is due to the polymers, which are osmotically less active and fermented by the microbiota, producing gas [46,47]. The poor absorption of FODMAPS is related to the absence or the reduced activity of luminal and/or brush border enzymes [48].

### 4.4. Wheat Germ Agglutinin (WGA)

The lectin wheat germ agglutinin is an additional important protein contained in wheat. Lectins are molecules normally produced by plants as a defense mechanism against fungi or other plants [49]. Interestingly, WGA induced an increase of epithelial permeability when administrated to Caco-2 cells, enhancing the passage of small molecules through the barrier [50].

In addition, WGA has an important effect on the immune system, as it can stimulate the immune system inducing the production of pro-inflammatory cytokines both in human peripheral blood mononuclear cells (PBMC) [50,51] and in animals [52,53].

## 5. Non-Celiac Gluten Sensitivity

NCGS or gluten sensitivity is a condition characterized by intestinal and extra-intestinal symptoms related to the ingestion of gluten-containing foods in patients in whom CD and wheat allergy have been excluded [54].

### 5.1. Epidemiology

The prevalence of NCGS remains heterogeneous, poorly defined, and based on a limited number of studies. The major limitation of data reporting the prevalence of NCGS relates to the lack of reliable diagnostic biomarkers. Most studies evaluated the prevalence of NCGS based on self-reported diagnoses, which could be highly influenced by various factors, including cultural, dietary, and regional differences in the perception of symptoms. Although a correct estimate of the prevalence of NCGS could be achieved only with the application of double-blind controlled studies, this would be unfeasible on a large scale. With these limitations in mind, and based on self-reported data only, the prevalence of NCGS has been claimed to range between 0.5% and 15% [2,4,5,9,10,11,55]. The prevalence of NCGS is higher in the third to fourth decade of life and generally higher in women than in men [2,4], although, based on a single study, in children, the prevalence of NCGS was higher in males than in females [56].

### 5.2. Clinical Characteristics

Patients with NCGS complain of abdominal symptoms referable to the gastrointestinal tract as well as extra-intestinal symptoms [54,57]. Typically, symptoms appear soon or immediately after the ingestion of gluten-containing foods; they disappear/ameliorate after gluten withdrawal and reappear after gluten challenge [54,58]. NCGS symptoms widely overlap with those reported by patients suffering from IBS and functional dyspepsia. Indeed, the most common symptoms reported by patients with NCGS are abdominal pain, bloating, and changes in bowel habits [54]. The most common extra-intestinal symptoms include tiredness, headache, foggy mind, anxiety, musculoskeletal, and skin manifestations, which are also common in patients with DGBI [54,57].

### 5.3. Diagnosis

In the absence of reliable biomarkers, the symptom overlaps between NCGS and other DGBI represents a diagnostic challenge in the differentiation of these entities and in the indication of dietary approaches including the reduction/avoidance of gluten-containing foods. The Salerno consensus conference proposed a diagnostic procedure based on a DBPC crossover gluten challenge [54]. Although this may represent the only reliable methodology allowing the identification of patients with gluten sensitivity, it remains cumbersome and inapplicable in daily clinical practice. A systematic review evaluated symptom relapse following the reintroduction of gluten-containing diet (GCD) in patients with NCGS diagnosed according to the Salerno criteria. Only three studies were eligible, and the results showed that symptom relapse was significantly higher in patients on a GCD compared to placebo [59]. A systematic review and meta-analysis evaluated 10 studies using a DBPC gluten challenge, irrespective of the amount of gluten and duration of gluten re-challenge, and it showed that symptoms related to gluten were present only in 16% of patients. In addition, a strong nocebo effect emerged in 40% of patients [60]. Heterogeneity in the amount of gluten used for the challenge, differences in the duration of re-challenge, and the lack of unambiguous criteria for diagnosis makes the comparison of published results difficult. This suggests that the identification of a reliable biomarker would help to better define the prevalence of NCGS and the appropriate identification of patients requiring gluten exclusion diets.

In the effort to find a biomarker of NCGS, Volta et al. assessed the prevalence of immunoglobulin G (IgG) anti-gliadin antibodies (AGA). Positive AGA–IgG antibodies were detected in 25 to 50% of patients with self-reported NCGS. Although interesting, these studies were uncontrolled, as no data were available in healthy subjects [61,62]. Uhde et al. demonstrated increased levels of the fatty acid-binding protein 2 (FABP2), suggesting a lack of barrier integrity [63], which is in line with other data showing permeability alterations in intestinal explants of NCGS patients [64]. In contrast, the evaluation of in vivo permeability by lactulose/mannitol test resulted to be normal [65].

We have recently demonstrated that the combination of zonulin serum levels, the endogenous modulator of permeability, with a combination of key symptoms and gender, can differentiate NCGS from irritable bowel syndrome with predominant diarrhea (IBS-D) with a diagnostic accuracy of 89% [66]. The study involved 59 patients with IBS-D, 86 patients with NCGS, either self-reported or confirmed in a double-blind fashion, 15 patients with CD, and 25 healthy subjects. Irritable bowel syndrome with predominant constipation (IBS-C) patients were not enrolled in this study, and it would be of interest to evaluate zonulin levels also in this subgroup of IBS patients in future studies. Zonulin serum levels were significantly increased both in NCGS and CD patients compared to healthy subjects and IBS-D patients. No differences emerged between NCGS and CD patients. Logistic regression was used to identify the variables to be included in a diagnostic algorithm to distinguish NCGS from IBS. The first step was the exclusion of CD and then the algorithm, which was based on zonulin serum levels, the severity of abdominal pain and distension, and gender; by a mathematical formula, we calculated an index (NvI): values of the index <1 were associated with a diagnosis of IBS-D, while values >1 were associated with NCGS. [66]. These results were obtained in a small sample of patients and need further validation. In addition, it cannot be excluded that the commercial kit used to detect zonulin can identify also some analogous proteins [67,68]. Accordingly, zonulin belongs to a family of structurally and functionally related proteins, which are named “zonulin family peptides” (ZFP), and it cannot be excluded that the commercially available enzyme-linked immunosorbent assay (ELISA) kit can detect different members of the ZFP, which can have effects on intestinal permeability [69].

### 5.4. Pathophysiology

The scanty information available on the pathophysiology of NCGS has hampered our understanding on the mechanisms underlying symptom generation. In the last few years, important progress has been made in understanding NCGS, and new data emerged supporting a role for intestinal barrier dysfunction, microbial alterations, and innate immune activation in this condition. Most available studies have focused their attention on the activation of the immune system. Although at endoscopic and histological examination intestinal morphology is unremarkable, up to 40% of patients with NCGS showed a mild increase in the number of intraepithelial lymphocytes compatible with Marsh 1 category according to the Oberhuner–Marsh classification [61]. Compared to controls and CD, patients with NCGS show a reduced expression of FOXP3, which is a marker of T-regulatory cells [65]. One study evaluated the effect of the gluten challenge on cytokines production by the duodenal biopsies in patients with NCGS and CD. The gluten challenge (four slices of gluten-containing bread daily for 3 days) increased the expression of interferon (IFN)-γ only in the intestinal mucosa of patients with NCGS [70]; interestingly, a role for IFN-γ was also reported in mucosal biopsies of patients with IBS [71], suggesting that this immunological profile represents a non-specific response for NCGS and rather a common immunological pathway involved in different gastrointestinal disorders. Recent work demonstrated that both the duodenal and the rectal mucosa of NCGS patients were characterized by an increased number of CD3+ cells, CD45+ cells, and eosinophils compared to non-NCGS patients [72]. Picarelli et al. reported a linear distribution of lymphocytes at the base of the mucosa in patients with NCGS, together with a slight increase of eosinophils in the lamina propria and increased circulating anti-gliadin (AGA) IgG [73]. The stratification of AGA IgG in subclasses showed that NCGS patients are characterized by a significant increase of IgG4 compared with CD and healthy cohorts, and IgG2 compared with healthy subjects [74]. Altogether, these data suggest that the intestine of NCGS is characterized by activation of the innate and adaptive immune response and mucosal inflammation.

A recent study evaluated neuronal and mast cell density and their vicinity in the submucosa of patients with self-reported NCGS, CD, and functional dyspepsia in comparison with healthy subjects. The number of neurons was not different among the groups, while mast cell density was significantly decreased in NCGS compared to patients with functional dyspepsia. Concerning the vicinity of mast cells to neurons, all three pathological groups showed a significant increase compared to healthy controls. Only in the group of NCGS was mast cell density correlated with abdominal pain severity and the percentage of mast cells close to neurons correlated with the severity of abdominal pain and bloating and with the number of gastrointestinal symptoms. This study did not clearly designate what proportion of their NCGS cohort qualified as being IBS according to the Rome criteria [75].

Some data suggest that gut microbiota may have implications for the activation of innate immune responses in the gut of NCGS patients. First, microbiota composition has been reported to differ in NCGS compared to healthy subjects and CD [40,76]. Second, mucosal biopsies of NCGS patients were characterized by an increased expression of Toll-like receptor (TLR)-2 compared to CD. TLR-2 is a transmembrane receptor that via its extracellular domains recognizes a large variety of Gram-positive and Gram-negative bacterial antigens. Third, the involvement of systemic immune activation to microbial components and microbiota translocation have also been described based on the evidence that patients with NCGS are characterized by significantly increased serum levels of soluble CD14, lipopolysaccharide-binding protein, and anti-bacterial antibodies [63]. In addition, this study demonstrated significant correlations between the concentration of FABP2, a marker of epithelial dysfunction, and serum levels of both lipopolysaccharide-binding protein and soluble CD14, which are markers of systemic immune activation, though the authors did not exclude IBS during the recruitment [63].

We recently reported that six-month wheat avoidance significantly reduced zonulin serum levels in NCGS patients carrying the HLA-DQ2/8 genotype. Zonulin, the pre-haptoglobin 2, is the only known endogenous modulator of intestinal permeability. These results suggested a role for permeability disfunction in the pathophysiology of NCGS. In addition, we found a correlation between zonulin and abdominal pain, distension, and extraintestinal symptoms in HLA-DQ2/-positive NCGS patients [66].

**Figure 2:**
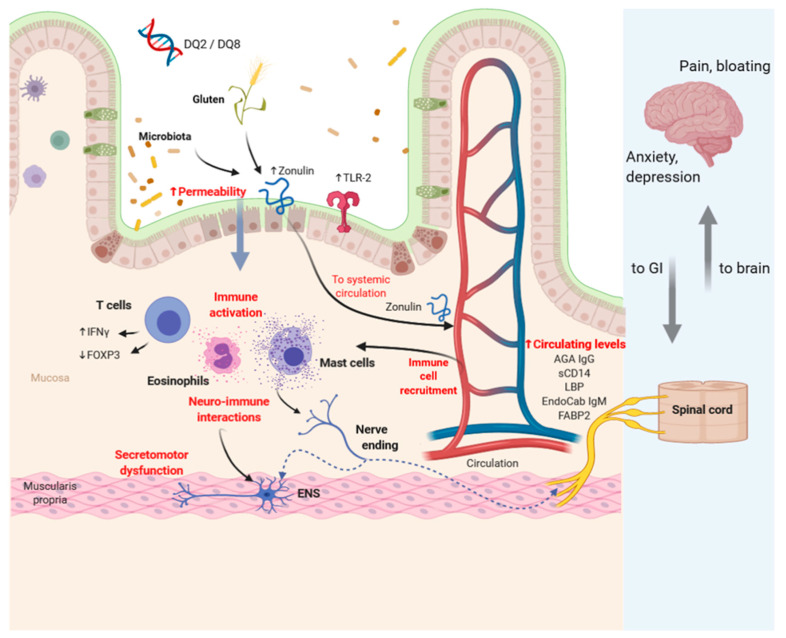
Representation of the potential mechanisms involved in the pathophysiology of non-celiac gluten sensitivity (NCGS). Based on the available data, the physiopathology of NCGS involves immune activation, permeability alteration, neuro-immune interactions, and genetic factors. Gluten and microbiota can increase epithelial permeability favoring the passage of different antigens into the mucosa and the consequent immune system activation. Neuro-immune activation and secretomotor dysfunction can likely (dashed lines) influence brain activity. TLR-2: Toll-like receptor-2; IFN-γ: interferon-γ; FOXP3: forkhead box P3; AGA IgG: anti-gliadin antibody IgG; sCD14: soluble CD14; LBP: lipopolysaccharide-binding protein; EndoCab IgM: endotoxin core antibodies IgM; FABP2: fatty acid-binding protein2; ENS: enteric nervous system; GI: gastrointestinal.

Gene expression was evaluated in the duodenal mucosa of 21 NCGS patients and 7 control subjects using microarray analysis. The results of this exploratory study showed 300 transcripts differently expressed in NCGS patients compared to controls; interestingly, most of these transcripts were non-coding RNAs. The protein-coding RNA differently expressed in the NCGS group was related to inflammation and immune response. Unfortunately, these data belong to a small number of patients; in addition, they were not validated in an external cohort, and NCGS patients were diagnosed according to an open gluten challenge [77]. For all these reasons, these preliminary results need to be confirmed in future studies. Figure 2 is a representation of the potential mechanisms involved in the pathophysiology of NCGS based on recent data.

Some studies suggest that gluten may evoke a wide range of intestinal and extra-intestinal symptoms. In 2015, Di Sabatino et al. conducted a DBPC crossover trial in 61 self-reported NCGS patients. This study demonstrated that a challenge with 4.375 g of gluten determined higher levels of symptoms compared with placebo challenge [78]. A multicenter trial involving 98 adults with functional gastrointestinal symptoms showed that 14% of these patients had NCGS as demonstrated by a placebo-controlled challenge with 5.6 g/day of gluten [79]. In a pediatric study involving 1.114 children complaining of functional gastrointestinal symptoms, 36 patients showed a stringent correlation between symptoms and gluten. They were further included in a DBPC trial with a gluten challenge of 10 g/day. Eleven of these 36 patients (39%) tested positive to the challenge and were diagnosed as NCGS [80].

Peters et al. evaluated the effect of gluten on mental state (depression, anxiety, anger, and curiosity) in a randomized, placebo-controlled, double-blind, crossover, gluten (16 g/day) challenge study in 22 self-reported NCGS patients. In addition, cortisol secretion and intestinal symptoms were recorded. Results showed decreased depression scores during GFD, although there was a persistence of gastrointestinal symptoms. No difference emerged in the other parameters evaluated. Although the study did not explore molecular mechanisms underlying this effect, a role for gut microbiota cannot be excluded [81].

Despite this above-mentioned evidence, there are contradictory data that question the role of gluten as a trigger of NCGS [82]. To assess whether symptoms were triggered by gluten in patients diagnosed as NCGS, Zanini et al. performed a double-blind challenge study involving 35 subjects with non-celiac disease. These patients were randomized to receive either a gluten-containing or non-containing flour for a period of 10 days. Then, the patients were subjected to a period of two weeks wash out and then crossed over to receive the alternative treatment. Only 34% (one-third) of patients correctly recognized gluten-containing flour. In addition, these patients reported higher symptom scores following gluten challenge compared to baseline and were diagnosed as NCGS [83]. A randomized DBPC crossover study investigated the role of gluten (5.7 g) and fructans (2.1 g) as symptom triggers in self-reported NCGS patients. The study envisaged a period of treatment of 7 days with gluten, fructans, or placebo, which was followed by wash out and crossover. The results showed that symptom scores during gluten treatment were significantly lower than during fructans consumption and similar to placebo [84].

A recent study evaluated the effect of a low-gliadin bread in patients with NCGS compared to gluten-free bread. The results demonstrated that this dietary intervention was effective in increasing the abundance of bacteria, including butyrate-producing ones, which are likely involved in the maintenance and improvement of gut permeability [85].

Roncoroni et al. investigated the level of tolerance to gluten in NCGS patients. Twenty-four NCGS patients, diagnosed according to the Salerno criteria, were enrolled. The study evaluated the effect of low (3.5–4 g/day), middle (6.7–8 g/day), or high (10–13 g/day) gluten reintroduction on symptoms and well-being after at least 3-weeks of GFD. The reintroduction of a low-gluten diet was associated with a decrease in the general well-being and quality of life, while higher doses of gluten were well tolerated. Studies involving a larger number of patients will be necessary to confirm these preliminary data [86]. A summary of the clinical studies that evaluated the effect of gluten re-challenge in NCGS patients after GFD is reported in Table 1.

The participation of gluten to symptom generation has been often proposed, but as of yet, it remains not proven with certainty. As wheat contains components other than gluten, other factors (i.e., FODMAPs and ATIs) have been called into play, hence inducing some authors to prefer the term non-celiac wheat sensitivity [87,88].

A double-blind crossover trial of 37 subjects with NCGS and IBS evaluated the effect of gluten after a reduced FODMAP diet. The results showed that the low FODMAP diet significantly reduced symptoms, while gluten (both high and low-gluten consumption) worsened them in the same way as whey proteins (considered as control diet) did. Only a small percentage (8%) of participants reported symptoms specifically related to gluten intake. Based on this evidence, the authors suggested a role for FODMAPs in the pathophysiology of NCGS and symptom generation [89].

In addition, a low FODMAP diet administered for two weeks improved significantly intestinal and extra-intestinal symptoms in patients with self-reported NCGS. Interestingly, GFD induced a more pronounced symptom reduction compared to the low-FODMAP diet [40]. As foods with gluten often contain fructans as a consequence, fructan intake is reduced in a low FODMAP diet as well as in a GFD, generating confusion in the interpretation of the studies [90]. A recent randomized placebo-controlled challenge study assessed the relative importance of these carbohydrates and gluten on symptoms in 59 individuals on a GFD for NCGS. The results showed that placebo, fructans, and gluten worsened symptoms; however, the relative effect of fructans was significantly higher than that of gluten [84]. Based on these results, the authors doubted the need for a GFD in individuals that self-report gluten sensitivity and suggest rather a low FODMAP diet [84]. The observed benefit of fructans on symptoms is likely via a non-immunogenic mechanism.

Noteworthy, a low-FODMAP diet reduced depressive symptoms in patients with NCGS [40] and CD [91] already on a GFD, suggesting that gluten is not the only modulator.

The hypothesis of a role for other wheat components other than gluten in NCGS symptoms generation was suggested by Dale et al. They performed a randomized, double-blind placebo-controlled challenge to evaluate the effect of gluten in patients with suspected NCGS. Only four out of 20 patients (20%) correctly recognized a gluten-containing muffin compared to a placebo due to a reduction of symptoms and were diagnosed as NCGS. No significant difference emerged in symptomatic score comparing gluten and placebo in the diagnosed NCGS nor the other patients. The authors suggested that suspected NCGS patients are affected by functional gastrointestinal disorders and their symptoms are provoked by diet, especially FODMAPS. Finally, a strong nocebo effect emerged from these results [92].

## 6. Overlap between Gluten Sensitivity and Disorders of Gut–Brain Interaction

Functional gastrointestinal disorders, now defined as disorders of gut–brain interaction (DGBI), encompass a group of conditions affecting the gastrointestinal tract, which are classified by gastrointestinal symptoms related to any combination of motility disturbance, visceral hypersensitivity, altered mucosal and immune function, gut dysbiosis, and altered central nervous system (CNS) processing of information conveyed from the periphery to the CNS [95]. Initial data suggest that NCGS shares some of these pathophysiological mechanisms.

### 6.1. Symptoms of DGBI in Patients with NCGS

A key question is whether patients with NCGS experience symptoms fulfilling criteria for IBS or other DGBI, principally functional dyspepsia (FD). A study conducted in the UK assessing the population prevalence of self-reported gluten sensitivity found that subjects with gluten sensitivity have a higher rate of fulfilment of Rome III criteria for IBS in comparison to those without gluten sensitivity (i.e., 20% versus 3.89%; *p* < 0.0001) [7]. Carroccio et al. performed a study in a group of Italian students aged between 14 and 19 years, showing that the frequency of self-reported non-celiac wheat sensitivity was 12.2%. Interestingly, 44% of subjects with self-reported non-celiac wheat sensitivity referred gastrointestinal symptoms fulfilling criteria for IBS diagnosis as compared with 25% of control subjects, with an odds ratio of 2.3. However, the criteria used for IBS diagnosis was not reported, making comparison of these data with those of other studies difficult. In addition, only a small proportion of subjects (2.9%) reported being on a gluten-free diet, suggesting that a “formal” diagnosis of non-celiac wheat sensitivity could not be performed in this epidemiological study [9].

A prospective multicenter survey in 486 Italian patients with NCGS reported a high prevalence of IBS and functional dyspepsia symptoms, including bloating (87%), abdominal pain (83%), diarrhea (54%), epigastric pain (52%), nausea (44%), alternating bowel habits (27%), and constipation (23%) [61]. An Italian randomized DBPC crossover trial reported a prevalence of 64.3% of IBS and 14.3% of FD in the group of confirmed NCGS patients [79].

In another cross-sectional prevalence study performed on a total of 3542 people randomly selected from the Australian population, the prevalence of self-reported wheat sensitivity was 14.9%. In addition, 45.3% of wheat sensitivity patients fulfilled criteria for a functional gastrointestinal disorder (either IBS or FD). Interestingly, in a multivariate analysis, self-reported wheat sensitivity was independently associated with Rome III IBS (odds ratio 3.55) and FD (odds ratio 1.48). The same study showed a prevalence of FD in self-reported wheat sensitivity patients of 31.3%. Among the FD-type symptoms, the study showed that 22.5% of patients referred postprandial fullness, 13.5% heartburn, 11.3% early satiety, and 7.4% nausea. The prevalence of IBS symptoms was the following: 54.3% of patients involved in the study reported abdominal pain, relieved with bowel motions, 36.9% reported bloating, 30.7% reported abdominal distention, 22.6% reported loose or watery bowel motions, and 16.8% reported hard or lumpy stool [10].

Recently, an Australian longitudinal study assessed the prevalence of self-reported non-celiac wheat sensitivity in 2015 and 2018 as well as the incidence and resolution of this condition. The overall prevalence of self-reported non-celiac wheat sensitivity according to the 2015 and 2018 surveys was similar and about 14%, while in 2018 a similar proportion of respondents, about 5.5%, reported new wheat sensitivity or resolution of this condition. At baseline (2015), based on a modified version of Rome III criteria, the prevalence of IBS was 32% in subjects with self-reported non-celiac wheat sensitivity as compared with 10.7% of the control group (odds ratio of 3.50), while the prevalence of functional dyspepsia (FD) was 27% but not significantly different from that of the control group. In contrast, the factors associated with new wheat sensitivity were younger age, female sex, and having FD. All together, these data suggest that IBS was strongly associated with baseline (2015), while FD was associated with incident (2018) self-reported non-celiac wheat sensitivity [11].

FD patients commonly complain symptoms related to food ingestion, including gluten and FODMAPs [96], and dietary intervention can be used to treat these patients [97].

A randomized double-blind placebo-controlled trial in patients with refractory FD demonstrated that GFD was effective in improving gastrointestinal symptoms in 35% of patients; after blind gluten challenge, symptoms recurred in 18% of GFD responders. This last group of patients was considered confirmed NCGS [98].

**Figure 3:**
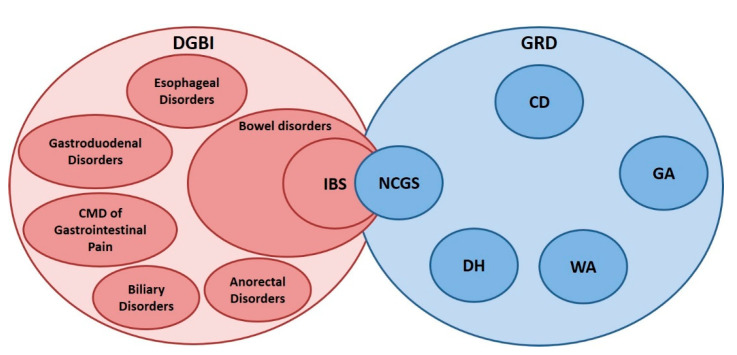
The overlap between IBS and NCGS. Disorders of gut–brain interaction (DGBI) include several disorders [95] affecting the gastrointestinal tract in which the gut–brain axis has a role in symptom generation. Among gluten-related disorders (GRD), NCGS is completely distinct from the other conditions including celiac disease (CD), gluten ataxia (GA), dermatitis herpetiform (DH), and wheat allergy (WA). NCGS is partially overlapped to IBS for symptoms, although there are also some differences. NCGS is characterized by a greater incidence, although not to the same extent as CD, of anemia, weight loss, low body mass index, atopy, osteopenia and osteoporosis compared to IBS. CMD of Gastrointestinal Pain: Centrally Mediated Disorders of Gastrointestinal Pain.

Based on data available in the literature, the prevalence of IBS in subjects with self-reported NCGS ranged from 20% to 44% as compared with 3.9% to 25% of the control group, suggesting an epidemiological association between IBS and self-reported NCGS (Figure 3). More controversial are the data assessing the association between functional dyspepsia and self-reported NCGS. If IBS is also associated with confirmed NCGS, this is virtually unknown and should be demonstrated in ad hoc studies.

### 6.2. NCGS among Patients Diagnosed as IBS

A different issue is whether a proportion of patients with IBS have an underrecognized NCGS. Even with the strict application of the current Rome IV criteria, the identification of a self-reported NCGS cannot be obtained, as questions regarding dietary habits are not included in the diagnostic process for IBS. A recent survey indicates that up to eight out of ten patients with IBS believed that their symptoms were related to food items. One-quarter of these patients self-reported that wheat-containing products were associated with symptom onset or aggravation and many of them decided to adopt a GFD [1,99]. Interestingly, the introduction of a GFD in a group of IBS-D patients who had never consider gluten as the cause of their symptoms and did not adopt a dietary approach was effective in reducing their symptom score. Seventy-two percent of these patients decided to adopt a GFD, and after a follow-up of 18 months, they still followed a GFD to control their symptoms [100]. In a randomized DBPC trial in patients with IBS, Biesiekierski et al. found that gluten significantly worsened overall symptoms, abdominal pain, abdominal bloating, tiredness, and satisfaction with stool consistency. No relationship was found between symptom improvement and HLA genotype typically associated with CD [93]. Some data suggest that in the effort to distinguish patients with NCGS from IBS, it may be worth assessing anemia [57], weight loss [57], low body mass index, atopy [57], osteopenia, and osteoporosis [101], as they have a higher frequency in NCGS, although not to the same extent as in CD [7] compared to IBS. Anti-gliadin AGA–IgG antibodies [61] have been reported in higher prevalence in NCGS, although the corresponding data in the healthy population were not evaluated. A recent paper reported that 22% of IBS patients were AGA positive [102]. In a randomized DBPC gluten re-challenge trial in patients with IBS, a GCD was associated with higher small bowel permeability, as assessed with the lactulose/mannitol test. Increased intestinal permeability was greater in HLA-DQ2/8-positive than HLA-DQ2/8-negative patients. In addition, patients on GCD had decreased mucosal expression of zonula occludens 1 in the mucosa of the small bowel and rectosigmoid region as well as decreased expression of claudin-1 and occludin in the rectosigmoid mucosa; the effects of the GCD on expression were significantly greater in HLA-DQ2/8-positive patients [103]. In line with these data, Shahbazkhani et al. performed a randomized DBPC trial in 72 IBS patients, showing a significant improvement of the overall symptoms during placebo compared to GCD (83.8% vs. 25.7% respectively) [94].

Using confocal laser endomicroscopy for the real-time visualization of structural/functional changes in the intestinal mucosa after food challenge, Fritscher-Ravens et al. identified that several food triggers reported by IBS-D patients, for whom allergy tests resulted negative, evoked rapid mucosal changes indicative of increased mucosal permeability and low-grade inflammation. Wheat challenge to the duodenal mucosa induced an increase in intraepithelial lymphocytes, epithelial breaks, and inter-villous spaces [104]. Based on this background, a recent study proposed a protocol to produce bread and pasta with reduced-gluten content and to evaluate the impact of these products on IBS symptoms. Foods with reduced gluten content were comparable to normal products for structure and taste, but were associated with less symptom scores and higher quality of life [105]. Although these data suggest that gluten and wheat exert a detrimental effect in IBS, several issues remain controversial. The efficacy of exclusion diets in IBS was evaluated in a systematic review of randomized controlled trials. GFD was evaluated in two out of nine randomized eligible studies and resulted to be associated with a reduction of global symptoms compared with a control diet (relative risk = 0.42), although the results did not reach statistical significance. The other seven studies evaluated the efficacy of a low-FODMAP diet compared with different control interventions; only three of these studies used rigorous control diets and showed a minimum effect. The authors concluded that the available data are insufficient to recommend a GFD in patients with IBS [106]. A recent paper evaluated the effect of GFD in IBS and looked for associated biomarkers. The results demonstrated that GFD improved symptoms in 75% of AGA positive IBS patients and 38% of AGA negative ones. In particular, the presence of anti-gliadin IgG was associated with a reduction in overall symptoms and in particular diarrhea. Based on these results, the authors proposed this class of antibody as a biomarker to identify IBS patients in whom GFD might improve symptoms. Future studies involving a higher number of patients are needed to confirm these preliminary results [102].

Recently, Barone et al. conducted a randomized DBPC crossover trial in patients with IBS to identify patients with NCGS. Forty-two IBS patients, diagnosed according to Rome IV criteria, entered the study and followed an open low FODMAP–GFD. Sixty-five percent of these patients reported symptom improvement defined as a reduction ≥30% of the visual analogue scale (VAS) score. These patients entered the DBPC crossover trial. Forty-six percent of patients reported an increase of VAS score ≥30% during gluten treatment compared to placebo phase and were classified as NCGS. Interestingly, the application of different criteria to evaluate symptoms improvement, such as that reported by Di Sabatino et al. [78], dramatically decreased the percentage of NCGS patients identified to 3.8% (i.e., only one patient). The authors concluded that a low FODMAP–GFD before gluten/placebo challenge is a better protocol to identify NCGS in IBS patients; however, the study did not test patients for CD and did not include the arm evaluating FODMAP reintroduction [107].

Rifaximin, a poorly absorbable antibiotic, improves abdominal pain and stool consistency in patients with IBS-D with a favorable safety profile [108]. A recent review of the literature suggested that rifaximin modulating gut microbiota may lead to a decrease in bacterial fermentation and a reduction of symptoms [109]. A double-blinded randomized study, involving 16 healthy volunteers who received either rifaximin (600 mg/day) or placebo for 7 days, evaluated the brain activity using magnetoencephalography during a social stress situation. The results showed that rifaximin reduced stress effects [110]. The effect of rifaximin was evaluated in chronically stressed mice. These animals were characterized by anxiety and neophobia at central level and by an increase of Bacteroidetes and Proteobacteria, principally Clostridium species, in the gut microbiota. The treatment with rifaximin decreased Clostridium concentration and lipopolysaccharide concentration in the plasma, and it reinforced the gut epithelial barrier without influencing the behavior [111].

## 7. Conclusions

The number of people avoiding gluten is increasing even in cases not affected by CD. An increasing proportion of subjects avoid gluten to improve gastrointestinal symptoms, which in some cases fulfill criteria for IBS. Initial evidence suggests a role for gluten in mood and psychiatric disorders [112,113,114], immune system activation [63], altered epithelial barrier [64], dysbiosis [40], and microbiota translocation [63] in NCGS patients. Clearly, many mechanisms involved in NCGS pathophysiology overlap with those of DGBI, although it is also possible that independent mechanisms exist. Nonetheless, although the diagnosis of NCGS is still a challenge, important progress has been made in understanding NCGS and in the development of potential biomarkers. Preclinical and clinical studies are now needed to definitively legitimize this condition, separate it from DGBI, and provide a clear indication for GFD.

## Figures and Tables

**Table 1:** A summary of the clinical studies that evaluated the effect of gluten re-challenge in NCGS patients after GFD. GCD, gluten-containing diet; GFD, gluten-free diet; NCGS, non-celiac gluten sensitivity; IBS, irritable bowel syndrome; CD, celiac disease; DBPC, double-blind placebo-controlled; FODMAP, fermentable oligosaccharides, disaccharides, monosaccharides and polyols; ATIs, α-amylase/trypsin inhibitors; GSRS, Gastrointestinal Symptom Rating Scale.

Study (Reference)	Patients	Study Design	No. of Patients	Methods	Main Findings	Limitations
**Biesiekierski et al. 2011 [93]**	NCGS w/IBS	Randomized DBPC trial	34 patients	Two bread slices plus one muffin per day with a GFD for up to 6 weeks	13/19 patients (68%) in the gluten group reported inadequate control of symptoms compared with 6/15 (40%) under placebo	Nocebo effect
**Carroccio et al. 2012 [57]**	NCGS	Randomized DBPC Crossover trial	920 patients	Capsules with wheat (20 g) vs. placebo for 2 consecutive weeks. After 1 week of wash out, patients received the other treatments for another 2 weeks	276 (30%) of patients diagnosed as non-celiac wheat sensitivity. DBPC challenge induced >30% increase in symptoms	This is a retrospective study and the data were not recorded according to a predesigned protocol
**Biesiekierski et al. 2013 [89]**	NCGS w/IBS symptoms	Randomized DBPC Crossover trial	37 patients	GFD for 6 weeks. Patients were randomly assigned to one of three diet treatments: high-gluten (16 g gluten/d), low-gluten (2 g gluten/d and 14 g whey protein/d), or control (16 g whey protein/d) diets for 1 week. After a wash out period of at least 2 weeks, participants crossed over to the next diet. Challenge duration: 5 weeks	Gluten-specific responses in 8% (three) of patients; 16% (six) of patients had an increase in overall GI symptoms in high gluten diet	High nocebo effect. Lack of association known from literature (i.e., between fatigue and gluten)
**Peters et al. 2014 [81]**	NCGS w/IBS symptoms	Randomized DBPC Crossover trial	22 patients	Low FODMAPs-GFD for 3 days. Challenge gluten-free food was supplemented with gluten (16 g/day), whey (16 g/day), or not supplemented (placebo) and administrated for 3 days. Wash out period between 3 and 14 days before crossover.	Gluten ingestion was associated with higher depression scores compared to placebo but not whey after gluten. Gastrointestinal symptoms were induced similarly by different dietary challenges	Small sample size. Restricted number of psychological end-points. Short dietary challenge to observe the maximum change in psychological states. Nocebo effect
**Zanini et al. 2015 [83]**	NCGS	Randomized DBPC Crossover	35 non-CD patients	Participants randomly received gluten-containing flour or not-containing flour for 10 days, followed by a wash out period of 2 weeks, and then crossed over to receive the alternative treatment	12 participants (34%) were classified as having NCGS	Some NCGS patients might be in an early “latent” stage of CD
**Shahbazkhani et al. 2015 [94]**	IBS	Randomized DBPC trial	72 patients	35/72 IBS patients were randomized in the gluten group, and 37/72 were in the placebo group. Patients previously following a strict GFD continued the gluten challenge for 6 weeks	Significant increase in GI symptoms after a gluten-containing meal challenge	Small sample size. Absence of crossover. High dose of gluten
**Di Sabatino et al. 2015 [78]**	Suspected NCGS	Randomized DBPC Crossover	61 patients	Participants followed a strict GFD before randomization to gluten or placebo for 1 week, each via gastro-soluble capsules. After 1 week of wash out, participants crossed over to the other group, for another week. After the second treatment week, all patients continued with their wash out from gluten. Challenge duration: 5 weeks	Gluten significantly increased overall symptoms (intestinal symptoms: abdominal bloating and pain; extra-intestinal symptoms: foggy mind, depression, aphthous stomatitis) compared with placebo group	Relatively short period of wash-out from gluten; the lack of a control group of non–gluten-sensitive subjects
**Picarelli et al. 2016 [73]**	NCGS	Randomized DBPC trial	26 patients	A gluten-containing croissant (10 g of gluten per croissant) randomly assigned to 13 patients and a gluten-free croissant to the other 13 patients. Challenge duration: 1 day	No difference in the severity of GI or extraintestinal symptoms between gluten intake and placebo	Small sample size
**Elli et al. 2016 [79]**	NCGS w/functional gastrointestinal symptoms	Randomized DBPC Crossover trial	98 patients	Patients were randomized to take gluten (5.6 g/day) or placebo for 7 days. Challenge duration: 21 days; 7 days on gluten or placebo, 7 days wash out, 7 days on gluten or placebo	28 patients showed symptomatic relapse during blind gluten ingestion with worsening of quality of life; 14 patients reported symptomatic worsening after placebo ingestion	Arbitrary gluten dosage and choice of timing. Missing evaluation of possible influence by other food constituents. Symptomatic deterioration was also observed in placebo group
**Skodje et al. 2018 [84]**	Subjects with self-reported NCGS	Randomized DBPC Crossover trial	59 subjects	Patients were randomized to follow diets containing gluten (5.7 g), fructans (2.1 g), or placebo, for 7 days. Following a minimum 7 days wash out, participants crossed over to next diet, until they completed all 3 challenges (gluten, fructan, and placebo)	Overall GSRS for IBS scores increased after fructans rather than gluten and placebo	High placebo response
**Dale et al. 2018 [92]**	Patients w/suspected NCGS	Randomized DBPC Crossover trial	20 patients	Two muffins a day (11/0 g gluten or placebo) for 4 days and wash out for 3 days. (4 periods of 4 days, 2 w/gluten and 2 w/placebo)	Most severe symptoms reported after placebo. Only 4/20 patients (20%) correctly identified periods w/gluten	Short wash-out period. Small sample size. Lack of control of confounding dietary. Timing of symptoms evaluation (in the morning) could be confounding
**Roncoroni et al. 2019 [86]**	NCGS	Increasing gluten amount	24 patients	GFD for 3 weeks, then patients received gradually increasing gluten diets: low-gluten diet (3.5–4 g gluten/day, week 1), mid-gluten diet (6.7–8 g gluten/day, week 2), and a high-gluten diet (10–13 g gluten/day, week 3). Patients w/o GI symptoms on a previous diet received more gluten-containing diet. Patients w/GI symptoms were shifted back to the previous-tolerated diet. Challenge duration: 6 weeks	Reintroduction of gluten in patients with NCGS who were on GFD induced different response: gluten at a low dosage induced a worsening of general well-being and the quality of life of a group of patients, whereas others tolerate even higher doses of dietary gluten	Small sample size. Absence of crossover

## References

[1] Bohn L., Storsrud S., Tornblom H., Bengtsson U., Simren M. (2013). Self-reported food-related gastrointestinal symptoms in IBS are common and associated with more severe symptoms and reduced quality of life. Am J. Gastroenterol..

[2] Cabrera-Chavez F., Dezar G.V., Islas-Zamorano A.P., Espinoza-Alderete J.G., Vergara-Jimenez M.J., Magana-Ordorica D., Ontiveros N. (2017). Prevalence of Self-Reported Gluten Sensitivity and Adherence to a Gluten-Free Diet in Argentinian Adult Population. Nutrients.

[3] Cabrera-Chavez F., Granda-Restrepo D.M., Aramburo-Galvez J.G., Franco-Aguilar A., Magana-Ordorica D., Vergara-Jimenez Mde J., Ontiveros N. (2016). Self-Reported Prevalence of Gluten-Related Disorders and Adherence to Gluten-Free Diet in Colombian Adult Population. Gastroenterol. Res. Pract..

[4] van Gils T., Nijeboer P., CE I.J., Sanders D.S., Mulder C.J., Bouma G. (2016). Prevalence and Characterization of Self-Reported Gluten Sensitivity in The Netherlands. Nutrients.

[5] Golley S., Corsini N., Topping D., Morell M., Mohr P. (2015). Motivations for avoiding wheat consumption in Australia: Results from a population survey. Public Health Nutr..

[6] Ontiveros N., Lopez-Gallardo J.A., Vergara-Jimenez M.J., Cabrera-Chavez F. (2015). Self-Reported Prevalence of Symptomatic Adverse Reactions to Gluten and Adherence to Gluten-Free Diet in an Adult Mexican Population. Nutrients.

[7] Aziz I., Lewis N.R., Hadjivassiliou M., Winfield S.N., Rugg N., Kelsall A., Newrick L., Sanders D.S. (2014). A UK study assessing the population prevalence of self-reported gluten sensitivity and referral characteristics to secondary care. Eur. J. Gastroenterol. Hepatol..

[8] Tanpowpong P., Ingham T.R., Lampshire P.K., Kirchberg F.F., Epton M.J., Crane J., Camargo C.A. (2012). Coeliac disease and gluten avoidance in New Zealand children. Arch. Dis. Child..

[9] Carroccio A., Giambalvo O., Blasca F., Iacobucci R., D’Alcamo A., Mansueto P. (2017). Self-Reported Non-Celiac Wheat Sensitivity in High School Students: Demographic and Clinical Characteristics. Nutrients.

[10] Potter M.D.E., Walker M.M., Jones M.P., Koloski N.A., Keely S., Talley N.J. (2018). Wheat Intolerance and Chronic Gastrointestinal Symptoms in an Australian Population-based Study: Association Between Wheat Sensitivity, Celiac Disease and Functional Gastrointestinal Disorders. Am. J. Gastroenterol..

[11] Potter M., Jones M.P., Walker M.M., Koloski N.A., Keely S., Holtmann G., Talley Ac N.J. (2020). Incidence and prevalence of self-reported non-coeliac wheat sensitivity and gluten avoidance in Australia. Med. J. Aust..

[12] Aziz I. (2018). The Global Phenomenon of Self-Reported Wheat Sensitivity. Am. J. Gastroenterol..

[13] Foschia M., Horstmann S., Arendt E.K., Zannini E. (2016). Nutritional therapy—Facing the gap between coeliac disease and gluten-free food. Int. J. Food Microbiol..

[14] Research G.V. Gluten-Free Products Market Size, Share & Trends Analysis Report by Product (Bakery Products, Dairy/Dairy Alternatives), by Distribution Channel (Grocery Stores, Mass Merchandiser), by Region, and Segment Forecasts, 2020–2027. https://www.grandviewresearch.com/industry-analysis/gluten-free-products-market.

[15] Dieterich W., Zopf Y. (2019). Gluten and FODMAPS-Sense of a Restriction/When Is Restriction Necessary?. Nutrients.

[16] Lerner B.A., Green P.H.R., Lebwohl B. (2019). Going against the Grains: Gluten-Free Diets in Patients without Celiac Disease-Worthwhile or Not?. Dig. Dis. Sci..

[17] The Hartman Group, Inc. Eating Gluten-Free. https://www.hartman-group.com/acumenPdfs/gluten-free-9_13_18.pdf.

[18] Lis D.M. (2019). Exit Gluten-Free and Enter Low FODMAPs: A Novel Dietary Strategy to Reduce Gastrointestinal Symptoms in Athletes. Sports Med..

[19] Croall I.D., Trott N., Rej A., Aziz I., O’Brien D.J., George H.A., Hossain M.Y., Marks L.J.S., Richardson J.I., Rigby R. (2019). A Population Survey of Dietary Attitudes towards Gluten. Nutrients.

[20] Potter M.D.E., Brienesse S.C., Walker M.M., Boyle A., Talley N.J. (2018). Effect of the gluten-free diet on cardiovascular risk factors in patients with coeliac disease: A systematic review. J. Gastroenterol. Hepatol..

[21] Hallert C., Grant C., Grehn S., Granno C., Hulten S., Midhagen G., Strom M., Svensson H., Valdimarsson T. (2002). Evidence of poor vitamin status in coeliac patients on a gluten-free diet for 10 years. Aliment. Pharmacol. Ther..

[22] Makovicky P., Makovicky P., Caja F., Rimarova K., Samasca G., Vannucci L. (2020). Celiac disease and gluten-free diet: Past, present, and future. Gastroenterol. Hepatol. Bed Bench.

[23] Bulka C.M., Davis M.A., Karagas M.R., Ahsan H., Argos M. (2017). The Unintended Consequences of a Gluten-free Diet. Epidemiology.

[24] Ludvigsson J.F., Lebwohl B., Chen Q., Broms G., Wolf R.L., Green P.H.R., Emilsson L. (2018). Anxiety after coeliac disease diagnosis predicts mucosal healing: A population-based study. Aliment. Pharmacol. Ther..

[25] Croall I.D., Aziz I., Trott N., Tosi P., Hoggard N., Sanders D.S. (2019). Gluten Does Not Induce Gastrointestinal Symptoms in Healthy Volunteers: A Double-Blind Randomized Placebo Trial. Gastroenterology.

[26] Spisni E., Imbesi V., Giovanardi E., Petrocelli G., Alvisi P., Valerii M.C. (2019). Differential Physiological Responses Elicited by Ancient and Heritage Wheat Cultivars Compared to Modern Ones. Nutrients.

[27] Prandi B., Tedeschi T., Folloni S., Galaverna G., Sforza S. (2017). Peptides from gluten digestion: A comparison between old and modern wheat varieties. Food Res. Int..

[28] Gianfrani C., Camarca A., Mazzarella G., Di Stasio L., Giardullo N., Ferranti P., Picariello G., Rotondi Aufiero V., Picascia S., Troncone R. (2015). Extensive in vitro gastrointestinal digestion markedly reduces the immune-toxicity of Triticum monococcum wheat: Implication for celiac disease. Mol. Nutr. Food Res..

[29] Ficco D.B.M., Prandi B., Amaretti A., Anfelli I., Leonardi A., Raimondi S., Pecchioni N., De Vita P., Faccini A., Sforza S. (2019). Comparison of gluten peptides and potential prebiotic carbohydrates in old and modern Triticum turgidum ssp. genotypes. Food Res. Int..

[30] Shewry P. (2019). What Is Gluten-Why Is It Special?. Front. Nutr..

[31] Balakireva A.V., Zamyatnin A.A. (2016). Properties of Gluten Intolerance: Gluten Structure, Evolution, Pathogenicity and Detoxification Capabilities. Nutrients.

[32] Silano M., Vincentini O., De Vincenzi M. (2009). Toxic, immunostimulatory and antagonist gluten peptides in celiac disease. Curr. Med. Chem..

[33] Picarelli A., Di Tola M., Sabbatella L., Anania M.C., Di Cello T., Greco R., Silano M., De Vincenzi M. (1999). 31-43 amino acid sequence of the alpha-gliadin induces anti-endomysial antibody production during in vitro challenge. Scand. J. Gastroenterol..

[34] Lammers K.M., Lu R., Brownley J., Lu B., Gerard C., Thomas K., Rallabhandi P., Shea-Donohue T., Tamiz A., Alkan S. (2008). Gliadin induces an increase in intestinal permeability and zonulin release by binding to the chemokine receptor CXCR3. Gastroenterology.

[35] Schuppan D. (2000). Current concepts of celiac disease pathogenesis. Gastroenterology.

[36] Maiuri L., Ciacci C., Ricciardelli I., Vacca L., Raia V., Auricchio S., Picard J., Osman M., Quaratino S., Londei M. (2003). Association between innate response to gliadin and activation of pathogenic T cells in coeliac disease. Lancet.

[37] Frossi B., Tripodo C., Guarnotta C., Carroccio A., De Carli M., De Carli S., Marino M., Calabro A., Pucillo C.E. (2017). Mast cells are associated with the onset and progression of celiac disease. J. Allergy Clin. Immunol..

[38] Andren Aronsson C., Lee H.S., Hard Af Segerstad E.M., Uusitalo U., Yang J., Koletzko S., Liu E., Kurppa K., Bingley P.J., Toppari J. (2019). Association of Gluten Intake During the First 5 Years of Life With Incidence of Celiac Disease Autoimmunity and Celiac Disease Among Children at Increased Risk. JAMA.

[39] Olivares M., Neef A., Castillejo G., Palma G.D., Varea V., Capilla A., Palau F., Nova E., Marcos A., Polanco I. (2015). The HLA-DQ2 genotype selects for early intestinal microbiota composition in infants at high risk of developing coeliac disease. Gut.

[40] Dieterich W., Schuppan D., Schink M., Schwappacher R., Wirtz S., Agaimy A., Neurath M.F., Zopf Y. (2019). Influence of low FODMAP and gluten-free diets on disease activity and intestinal microbiota in patients with non-celiac gluten sensitivity. Clin. Nutr..

[41] Bertini I., Calabro A., De Carli V., Luchinat C., Nepi S., Porfirio B., Renzi D., Saccenti E., Tenori L. (2009). The metabonomic signature of celiac disease. J. Proteome Res..

[42] Karakula-Juchnowicz H., Rog J., Juchnowicz D., Loniewski I., Skonieczna-Zydecka K., Krukow P., Futyma-Jedrzejewska M., Kaczmarczyk M. (2019). The study evaluating the effect of probiotic supplementation on the mental status, inflammation, and intestinal barrier in major depressive disorder patients using gluten-free or gluten-containing diet (SANGUT study): A 12-week, randomized, double-blind, and placebo-controlled clinical study protocol. Nutr. J..

[43] Junker Y., Zeissig S., Kim S.J., Barisani D., Wieser H., Leffler D.A., Zevallos V., Libermann T.A., Dillon S., Freitag T.L. (2012). Wheat amylase trypsin inhibitors drive intestinal inflammation via activation of toll-like receptor 4. J. Exp. Med..

[44] Zevallos V.F., Raker V., Tenzer S., Jimenez-Calvente C., Ashfaq-Khan M., Russel N., Pickert G., Schild H., Steinbrink K., Schuppan D. (2017). Nutritional Wheat Amylase-Trypsin Inhibitors Promote Intestinal Inflammation via Activation of Myeloid Cells. Gastroenterology.

[45] Spiller R. (2017). How do FODMAPs work?. J. Gastroenterol. Hepatol..

[46] Nanayakkara W.S., Skidmore P.M., O’Brien L., Wilkinson T.J., Gearry R.B. (2016). Efficacy of the low FODMAP diet for treating irritable bowel syndrome: The evidence to date. Clin. Exp. Gastroenterol..

[47] Barrett J.S., Gearry R.B., Muir J.G., Irving P.M., Rose R., Rosella O., Haines M.L., Shepherd S.J., Gibson P.R. (2010). Dietary poorly absorbed, short-chain carbohydrates increase delivery of water and fermentable substrates to the proximal colon. Aliment. Pharmacol. Ther..

[48] Magge S., Lembo A. (2012). Low-FODMAP Diet for Treatment of Irritable Bowel Syndrome. Gastroenterol. Hepatol..

[49] de Punder K., Pruimboom L. (2013). The dietary intake of wheat and other cereal grains and their role in inflammation. Nutrients.

[50] Dalla Pellegrina C., Perbellini O., Scupoli M.T., Tomelleri C., Zanetti C., Zoccatelli G., Fusi M., Peruffo A., Rizzi C., Chignola R. (2009). Effects of wheat germ agglutinin on human gastrointestinal epithelium: Insights from an experimental model of immune/epithelial cell interaction. Toxicol. Appl. Pharmacol..

[51] Haas H., Falcone F.H., Schramm G., Haisch K., Gibbs B.F., Klaucke J., Poppelmann M., Becker W.M., Gabius H.J., Schlaak M. (1999). Dietary lectins can induce in vitro release of IL-4 and IL-13 from human basophils. Eur. J. Immunol..

[52] Muraille E., Pajak B., Urbain J., Leo O. (1999). Carbohydrate-bearing cell surface receptors involved in innate immunity: Interleukin-12 induction by mitogenic and nonmitogenic lectins. Cell Immunol..

[53] Sodhi A., Kesherwani V. (2007). Production of TNF-alpha, IL-1beta, IL-12 and IFN-gamma in murine peritoneal macrophages on treatment with wheat germ agglutinin in vitro: Involvement of tyrosine kinase pathways. Glycoconj. J..

[54] Catassi C., Elli L., Bonaz B., Bouma G., Carroccio A., Castillejo G., Cellier C., Cristofori F., de Magistris L., Dolinsek J. (2015). Diagnosis of Non-Celiac Gluten Sensitivity (NCGS): The Salerno Experts’ Criteria. Nutrients.

[55] Capannolo A., Viscido A., Barkad M.A., Valerii G., Ciccone F., Melideo D., Frieri G., Latella G. (2015). Non-Celiac Gluten Sensitivity among Patients Perceiving Gluten-Related Symptoms. Digestion.

[56] Francavilla R., Cristofori F., Castellaneta S., Polloni C., Albano V., Dellatte S., Indrio F., Cavallo L., Catassi C. (2014). Clinical, serologic, and histologic features of gluten sensitivity in children. J. Pediatr..

[57] Carroccio A., Mansueto P., Iacono G., Soresi M., D’Alcamo A., Cavataio F., Brusca I., Florena A.M., Ambrosiano G., Seidita A. (2012). Non-celiac wheat sensitivity diagnosed by double-blind placebo-controlled challenge: Exploring a new clinical entity. Am. J. Gastroenterol..

[58] Sapone A., Bai J.C., Ciacci C., Dolinsek J., Green P.H., Hadjivassiliou M., Kaukinen K., Rostami K., Sanders D.S., Schumann M. (2012). Spectrum of gluten-related disorders: Consensus on new nomenclature and classification. BMC Med..

[59] Lionetti E., Pulvirenti A., Vallorani M., Catassi G., Verma A.K., Gatti S., Catassi C. (2017). Re-challenge Studies in Non-celiac Gluten Sensitivity: A Systematic Review and Meta-Analysis. Front. Physiol..

[60] Molina-Infante J., Carroccio A. (2017). Suspected Nonceliac Gluten Sensitivity Confirmed in Few Patients After Gluten Challenge in Double-Blind, Placebo-Controlled Trials. Clin. Gastroenterol. Hepatol..

[61] Volta U., Bardella M.T., Calabro A., Troncone R., Corazza G.R., The Study Group for Non-Celiac Gluten Sensitivity (2014). An Italian prospective multicenter survey on patients suspected of having non-celiac gluten sensitivity. BMC Med..

[62] Volta U., Tovoli F., Cicola R., Parisi C., Fabbri A., Piscaglia M., Fiorini E., Caio G. (2012). Serological tests in gluten sensitivity (nonceliac gluten intolerance). J. Clin. Gastroenterol..

[63] Uhde M., Ajamian M., Caio G., De Giorgio R., Indart A., Green P.H., Verna E.C., Volta U., Alaedini A. (2016). Intestinal cell damage and systemic immune activation in individuals reporting sensitivity to wheat in the absence of coeliac disease. Gut.

[64] Hollon J., Puppa E.L., Greenwald B., Goldberg E., Guerrerio A., Fasano A. (2015). Effect of gliadin on permeability of intestinal biopsy explants from celiac disease patients and patients with non-celiac gluten sensitivity. Nutrients.

[65] Sapone A., Lammers K.M., Casolaro V., Cammarota M., Giuliano M.T., De Rosa M., Stefanile R., Mazzarella G., Tolone C., Russo M.I. (2011). Divergence of gut permeability and mucosal immune gene expression in two gluten-associated conditions: Celiac disease and gluten sensitivity. BMC Med..

[66] Barbaro M.R., Cremon C., Morselli-Labate A.M., Di Sabatino A., Giuffrida P., Corazza G.R., Di Stefano M., Caio G., Latella G., Ciacci C. (2020). Serum zonulin and its diagnostic performance in non-coeliac gluten sensitivity. Gut.

[67] Ajamian M., Steer D., Rosella G., Gibson P.R. (2019). Serum zonulin as a marker of intestinal mucosal barrier function: May not be what it seems. PLoS ONE.

[68] Scheffler L., Crane A., Heyne H., Tonjes A., Schleinitz D., Ihling C.H., Stumvoll M., Freire R., Fiorentino M., Fasano A. (2018). Widely Used Commercial ELISA Does Not Detect Precursor of Haptoglobin2, but Recognizes Properdin as a Potential Second Member of the Zonulin Family. Front. Endocrinol..

[69] Fasano A. (2020). Zonulin measurement conundrum: Add confusion to confusion does not lead to clarity. Gut.

[70] Brottveit M., Beitnes A.C., Tollefsen S., Bratlie J.E., Jahnsen F.L., Johansen F.E., Sollid L.M., Lundin K.E. (2013). Mucosal cytokine response after short-term gluten challenge in celiac disease and non-celiac gluten sensitivity. Am. J. Gastroenterol..

[71] Barbaro M.R., Di Sabatino A., Cremon C., Giuffrida P., Fiorentino M., Altimari A., Bellacosa L., Stanghellini V., Barbara G. (2016). Interferon-gamma is increased in the gut of patients with irritable bowel syndrome and modulates serotonin metabolism. Am. J. Physiol. Gastrointest. Liver Physiol..

[72] Carroccio A., Giannone G., Mansueto P., Soresi M., La Blasca F., Fayer F., Iacobucci R., Porcasi R., Catalano T., Geraci G. (2019). Duodenal and Rectal Mucosa Inflammation in Patients with Non-celiac Wheat Sensitivity. Clin. Gastroenterol. Hepatol..

[73] Picarelli A., Borghini R., Di Tola M., Marino M., Urciuoli C., Isonne C., Puzzono M., Porowska B., Rumi G., Lonardi S. (2016). Intestinal, Systemic, and Oral Gluten-related Alterations in Patients with Nonceliac Gluten Sensitivity. J. Clin. Gastroenterol..

[74] Uhde M., Caio G., De Giorgio R., Green P.H., Volta U., Alaedini A. (2020). Subclass Profile of IgG Antibody Response to Gluten Differentiates Nonceliac Gluten Sensitivity From Celiac Disease. Gastroenterology.

[75] Giancola F., Volta U., Repossi R., Latorre R., Beeckmans D., Carbone F., Van den Houte K., Bianco F., Bonora E., Gori A. (2020). Mast cell-nerve interactions correlate with bloating and abdominal pain severity in patients with non-celiac gluten/wheat sensitivity. Neurogastroenterol. Motil..

[76] Garcia-Mazcorro J.F., Rivera-Gutierrez X., Cobos-Quevedo O.J., Grube-Pagola P., Meixueiro-Daza A., Hernandez-Flores K., Cabrera-Jorge F.J., Vivanco-Cid H., Dowd S.E., Remes-Troche J.M. (2018). First Insights into the Gut Microbiota of Mexican Patients with Celiac Disease and Non-Celiac Gluten Sensitivity. Nutrients.

[77] Efthymakis K., Clemente E., Marchioni M., Di Nicola M., Neri M., Sallese M. (2020). An Exploratory Gene Expression Study of the Intestinal Mucosa of Patients with Non-Celiac Wheat Sensitivity. Int. J. Mol. Sci..

[78] Di Sabatino A., Volta U., Salvatore C., Biancheri P., Caio G., De Giorgio R., Di Stefano M., Corazza G.R. (2015). Small Amounts of Gluten in Subjects With Suspected Nonceliac Gluten Sensitivity: A Randomized, Double-Blind, Placebo-Controlled, Cross-Over Trial. Clin. Gastroenterol. Hepatol..

[79] Elli L., Tomba C., Branchi F., Roncoroni L., Lombardo V., Bardella M.T., Ferretti F., Conte D., Valiante F., Fini L. (2016). Evidence for the Presence of Non-Celiac Gluten Sensitivity in Patients with Functional Gastrointestinal Symptoms: Results from a Multicenter Randomized Double-Blind Placebo-Controlled Gluten Challenge. Nutrients.

[80] Francavilla R., Cristofori F., Verzillo L., Gentile A., Castellaneta S., Polloni C., Giorgio V., Verduci E., D’Angelo E., Dellatte S. (2018). Randomized Double-Blind Placebo-Controlled Crossover Trial for the Diagnosis of Non-Celiac Gluten Sensitivity in Children. Am. J. Gastroenterol..

[81] Peters S.L., Biesiekierski J.R., Yelland G.W., Muir J.G., Gibson P.R. (2014). Randomised clinical trial: Gluten may cause depression in subjects with non-coeliac gluten sensitivity—An exploratory clinical study. Aliment. Pharmacol. Ther..

[82] Gibson P.R., Lundin K.E.A., Guandalini S. (2018). Is Non-Celiac Rice-Starch Sensitivity as Common in Children as Non-Celiac Gluten Sensitivity?. Am. J. Gastroenterol..

[83] Zanini B., Basche R., Ferraresi A., Ricci C., Lanzarotto F., Marullo M., Villanacci V., Hidalgo A., Lanzini A. (2015). Randomised clinical study: Gluten challenge induces symptom recurrence in only a minority of patients who meet clinical criteria for non-coeliac gluten sensitivity. Aliment. Pharmacol. Ther..

[84] Skodje G.I., Sarna V.K., Minelle I.H., Rolfsen K.L., Muir J.G., Gibson P.R., Veierod M.B., Henriksen C., Lundin K.E.A. (2018). Fructan, Rather Than Gluten, Induces Symptoms in Patients With Self-Reported Non-Celiac Gluten Sensitivity. Gastroenterology.

[85] Haro C., Villatoro M., Vaquero L., Pastor J., Gimenez M.J., Ozuna C.V., Sanchez-Leon S., Garcia-Molina M.D., Segura V., Comino I. (2018). The Dietary Intervention of Transgenic Low-Gliadin Wheat Bread in Patients with Non-Celiac Gluten Sensitivity (NCGS) Showed No Differences with Gluten Free Diet (GFD) but Provides Better Gut Microbiota Profile. Nutrients.

[86] Roncoroni L., Bascunan K.A., Vecchi M., Doneda L., Bardella M.T., Lombardo V., Scricciolo A., Branchi F., Elli L. (2019). Exposure to Different Amounts of Dietary Gluten in Patients with Non-Celiac Gluten Sensitivity (NCGS): An Exploratory Study. Nutrients.

[87] Carroccio A., Rini G., Mansueto P. (2014). Non-celiac wheat sensitivity is a more appropriate label than non-celiac gluten sensitivity. Gastroenterology.

[88] Tavakkoli A., Lewis S.K., Tennyson C.A., Lebwohl B., Green P.H. (2014). Characteristics of patients who avoid wheat and/or gluten in the absence of Celiac disease. Dig. Dis. Sci..

[89] Biesiekierski J.R., Peters S.L., Newnham E.D., Rosella O., Muir J.G., Gibson P.R. (2013). No effects of gluten in patients with self-reported non-celiac gluten sensitivity after dietary reduction of fermentable, poorly absorbed, short-chain carbohydrates. Gastroenterology.

[90] Rej A., Trott N., Aziz I., Sanders D.S. (2019). A Gluten-Free Diet: The Express Route to Fructan Reduction. Am. J. Gastroenterol..

[91] Roncoroni L., Bascunan K.A., Doneda L., Scricciolo A., Lombardo V., Branchi F., Ferretti F., Dell’Osso B., Montanari V., Bardella M.T. (2018). A Low FODMAP Gluten-Free Diet Improves Functional Gastrointestinal Disorders and Overall Mental Health of Celiac Disease Patients: A Randomized Controlled Trial. Nutrients.

[92] Dale H.F., Hatlebakk J.G., Hovdenak N., Ystad S.O., Lied G.A. (2018). The effect of a controlled gluten challenge in a group of patients with suspected non-coeliac gluten sensitivity: A randomized, double-blind placebo-controlled challenge. Neurogastroenterol. Motil..

[93] Biesiekierski J.R., Newnham E.D., Irving P.M., Barrett J.S., Haines M., Doecke J.D., Shepherd S.J., Muir J.G., Gibson P.R. (2011). Gluten causes gastrointestinal symptoms in subjects without celiac disease: A double-blind randomized placebo-controlled trial. Am. J. Gastroenterol..

[94] Shahbazkhani B., Sadeghi A., Malekzadeh R., Khatavi F., Etemadi M., Kalantri E., Rostami-Nejad M., Rostami K. (2015). Non-Celiac Gluten Sensitivity Has Narrowed the Spectrum of Irritable Bowel Syndrome: A Double-Blind Randomized Placebo-Controlled Trial. Nutrients.

[95] Drossman D.A., Hasler W.L. (2016). Rome IV-Functional GI Disorders: Disorders of Gut-Brain Interaction. Gastroenterology.

[96] Pesce M., Cargiolli M., Cassarano S., Polese B., De Conno B., Aurino L., Mancino N., Sarnelli G. (2020). Diet and functional dyspepsia: Clinical correlates and therapeutic perspectives. World J. Gastroenterol..

[97] Tan V.P. (2017). The low-FODMAP diet in the management of functional dyspepsia in East and Southeast Asia. J. Gastroenterol. Hepatol..

[98] Shahbazkhani B., Fanaeian M.M., Farahvash M.J., Aletaha N., Alborzi F., Elli L., Shahbazkhani A., Zebardast J., Rostami-Nejad M. (2020). Prevalence of Non-Celiac Gluten Sensitivity in Patients with Refractory Functional Dyspepsia: A Randomized Double-blind Placebo Controlled Trial. Sci. Rep..

[99] Monsbakken K.W., Vandvik P.O., Farup P.G. (2006). Perceived food intolerance in subjects with irritable bowel syndrome—Etiology, prevalence and consequences. Eur. J. Clin. Nutr..

[100] Aziz I., Trott N., Briggs R., North J.R., Hadjivassiliou M., Sanders D.S. (2016). Efficacy of a Gluten-Free Diet in Subjects With Irritable Bowel Syndrome-Diarrhea Unaware of Their HLA-DQ2/8 Genotype. Clin. Gastroenterol. Hepatol..

[101] Carroccio A., Soresi M., D’Alcamo A., Sciume C., Iacono G., Geraci G., Brusca I., Seidita A., Adragna F., Carta M. (2014). Risk of low bone mineral density and low body mass index in patients with non-celiac wheat-sensitivity: A prospective observation study. BMC Med..

[102] Pinto-Sanchez M.I., Nardelli A., Borojevic R., De Palma G., Causada Calo N., McCarville J., Caminero A., Basra D., Mordhorst A., Ignatova E. (2020). Gluten-free Diet Reduces Symptoms, Particularly Diarrhea, in Patients with Irritable Bowel Syndrome and Anti-gliadin IgG. Clin. Gastroenterol. Hepatol..

[103] Vazquez-Roque M.I., Camilleri M., Smyrk T., Murray J.A., Marietta E., O’Neill J., Carlson P., Lamsam J., Janzow D., Eckert D. (2013). A controlled trial of gluten-free diet in patients with irritable bowel syndrome-diarrhea: Effects on bowel frequency and intestinal function. Gastroenterology.

[104] Fritscher-Ravens A., Schuppan D., Ellrichmann M., Schoch S., Rocken C., Brasch J., Bethge J., Bottner M., Klose J., Milla P.J. (2014). Confocal endomicroscopy shows food-associated changes in the intestinal mucosa of patients with irritable bowel syndrome. Gastroenterology.

[105] Calasso M., Francavilla R., Cristofori F., De Angelis M., Gobbetti M. (2018). New Protocol for Production of Reduced-Gluten Wheat Bread and Pasta and Clinical Effect in Patients with Irritable Bowel Syndrome: A randomised, Double-Blind, Cross-Over Study. Nutrients.

[106] Dionne J., Ford A.C., Yuan Y., Chey W.D., Lacy B.E., Saito Y.A., Quigley E.M.M., Moayyedi P. (2018). A Systematic Review and Meta-Analysis Evaluating the Efficacy of a Gluten-Free Diet and a Low FODMAPs Diet in Treating Symptoms of Irritable Bowel Syndrome. Am. J. Gastroenterol..

[107] Barone M., Gemello E., Viggiani M.T., Cristofori F., Renna C., Iannone A., Di Leo A., Francavilla R. (2020). Evaluation of Non-Celiac Gluten Sensitivity in Patients with Previous Diagnosis of Irritable Bowel Syndrome: A Randomized Double-Blind Placebo-Controlled Crossover Trial. Nutrients.

[108] Cangemi D.J., Lacy B.E. (2019). Management of irritable bowel syndrome with diarrhea: A review of nonpharmacological and pharmacological interventions. Therap. Adv. Gastroenterol..

[109] Pimentel M. (2016). Review article: Potential mechanisms of action of rifaximin in the management of irritable bowel syndrome with diarrhoea. Aliment. Pharmacol. Ther..

[110] Wang H., Braun C., Enck P. (2018). Effects of Rifaximin on Central Responses to Social Stress-a Pilot Experiment. Neurotherapeutics.

[111] Kuti D., Winkler Z., Horvath K., Juhasz B., Paholcsek M., Stagel A., Gulyas G., Czegledi L., Ferenczi S., Kovacs K.J. (2019). Gastrointestinal (non-systemic) antibiotic rifaximin differentially affects chronic stress-induced changes in colon microbiome and gut permeability without effect on behavior. Brain Behav. Immun..

[112] Jackson J., Eaton W., Cascella N., Fasano A., Santora D., Sullivan K., Feldman S., Raley H., McMahon R.P., Carpenter W.T. (2014). Gluten sensitivity and relationship to psychiatric symptoms in people with schizophrenia. Schizophr. Res..

[113] Busby E., Bold J., Fellows L., Rostami K. (2018). Mood Disorders and Gluten: It’s Not All in Your Mind! A Systematic Review with Meta-Analysis. Nutrients.

[114] Doenyas C. (2018). Dietary interventions for autism spectrum disorder: New perspectives from the gut-brain axis. Physiol. Behav..

